# Fibre-Enriched Pasta from Wet Milled Royal Quinoa: Technological and Nutritional Characterisation

**DOI:** 10.3390/foods15081374

**Published:** 2026-04-15

**Authors:** Andrea Alonso-Álvarez, Claudia Monika Haros

**Affiliations:** Cereal Group, Instituto de Agroquímica y Tecnología de Alimentos (IATA-CSIC), Av. Agustín Escardino 7, Parque Científico, 46980 Paterna, Valencia, Spain; a.alonso@iata.csic.es

**Keywords:** *Chenopodium quinoa*, wet milling, quinoa fractionation, quinoa fibre, fresh pasta, nutritional value, mineral bioavailability

## Abstract

Quinoa fibre-rich fraction (QFi), obtained through wet milling, represents an innovative approach to improving the nutritional and functional quality of cereal-based products. Unlike conventional whole quinoa flour (WhQF), wet milling induces phytate losses during steeping, generating ingredients with enhanced mineral bioavailability. This study evaluated the incorporation of QFi into wheat pasta, assessing dietary fibre contribution, mineral bioavailability, cooking behaviour, and colour. Six fortified formulations were prepared by partially replacing wheat flour with WQF (white, red, or black) or QFi from the same varieties, with inclusion levels adjusted to provide equivalent dietary fibre across formulations. All quinoa-enriched pastas raised dietary fibre contribution compared with the control. Mineral contents also incremented, with the greatest values observed in formulations containing black quinoa ingredients. Fe and Zn contents were greatest in pastas with black WhQF, while Ca concentration was richer in formulations containing black QFi. Mineral absorption may be partially inhibited in pastas with WhQF, particularly in those containing the red quinoa. In contrast, QFi showed reduced phytate levels, highlighting the nutritional advantage of wet milling. Technologically, quinoa ingredients increased water absorption during pasta cooking. Overall, wet milled QFi provides a novel alternative to WhQF, combining improved mineral bioavailability with suitable technological properties for pasta processing.

## 1. Introduction

Quinoa (*Chenopodium quinoa* Willd.) has attracted increasing interest in food science for its exceptional nutritional profile, high-quality protein, and phytochemicals, as well as for its notable dietary fibre composition [1,2,3,4]. It is cultivated in a variety of seed colours, which reflect significant differences in the grain’s phytochemical content. Generally, darker-coloured quinoa seeds are richer in bioactive compounds such as betalains, flavonoids, and phenolic acids, which contribute to their antioxidant potential [5,6,7,8]. For instance, Ballester-Sánchez et al. [9] incorporated coloured quinoa flours (WhQFs) into wheat bread at a 25% substitution rate, leading to higher antioxidant capacity, particularly in breads made with red and black WhQFs, reaching approximately twofold (DPPH) and up to threefold (FRAP) compared to the control.

Beyond the use of WhQFs, recent advances have enabled the isolation of fibre-rich fraction (QFi) through wet milling, a process that separates starch, proteins, germ, and fibre [10,11]. This quinoa-derived fraction displays unique nutritional and techno-functional properties that distinguish them from traditional cereal fibres, combining a high total and insoluble fibre content with reduced phytic acid levels, potentially improving mineral bioavailability [10]. Their concentrated fibre content positions them as valuable ingredients to help address the widespread shortfall in dietary fibre intake [12], which remains below the 25 g/day recommended by the World Health Organization [13]. Black quinoa fibre (BQFi), in particular, shows elevated insoluble fibre, and favourable phytate-to-mineral ratios, suggesting improved Ca and Zn bioavailability compared with WhQFs [10].

Red quinoa fibre (RQFi) exhibits improved hydration and oil-holding capacities, which could be advantageous for texture and stability in food formulations. More broadly, QFi obtained through wet milling are characterised by a predominance of insoluble fibre and higher concentrations of certain minerals, while their reduced phytic acid content reflects phytate losses that probably occur during the steeping and separation steps of the wet milling process. This combination reinforces their potential as functional food ingredients with both nutritional and technological relevance [10]. Despite these promising attributes, their behaviour in real food matrices has not yet been fully explored. In particular, questions remain regarding their impact on product composition, colour, and techno-functional properties when integrated into commonly consumed staples. Pasta provides an ideal model system for such an evaluation, as it is widely consumed worldwide and frequently used as a vehicle for nutritional enrichment [14,15,16,17].

This study aimed to evaluate the impact of incorporating quinoa-derived ingredients into wheat-based pasta on their nutritional and functional qualities. Six fortified pasta formulations were prepared by partially replacing wheat flour (WF) with either 25% WhQF from white, red, or black varieties, or QFi obtained through wet milling from the same quinoa varieties, at inclusion levels calculated to match the dietary fibre content of the formulations containing WhQF. These formulations were compared to a control pasta made entirely with WF, with the aim of assessing the potential of the QFi to enhance the nutritional profile and functional properties of cereal-based foods.

## 2. Materials and Methods

### 2.1. Raw Materials

Wheat flour (medium-strength), sourced from Harinera La Meta S.A. (Lleida, Spain), was used in pasta production and presented the following proximate composition: 11.4 ± 0.3 g/100 g of moisture and, on a dry matter basis, 75.4 ± 0.7 g/100 g of starch; 11.4 ± 0.6 g/100 g of proteins; 1.34 ± 0.20 g/100 g of lipids; 0.600 ± 0.030 g/100 g of ashes; and 3.32 ± 0.32 g/100 g of total dietary fibre. This WF had a particle size below 0.150 mm. Royal quinoa grains of white, red, and black phenotypes, naturally occurring cultivars within the Quinoa Real landrace group, were obtained from the Organic Quinoa Real^®^ commercial brand (Uyuni, Bolivia); they were milled into WhQFs using an electric grinder (Aromatic, Taurus; Oliana, Spain) for use in pasta formulation. Grinding was performed for a total of 30 s (three cycles of 10 s each). The resulting WhQFs exhibited broader particle size distributions, ranging from 75 to 425 µm, with 60% of the particle volume between 100 and 300 µm. Egg yolks were manually separated from whole commercial eggs purchased locally.

### 2.2. Wet Milling Procedure and Dietary Fibre Isolation

Fibre-rich fraction was isolated from white, red, and black quinoa seeds through a laboratory-scale wet milling process [10]. Quinoa grains (100 g) were steeped in 1.0 L of sodium bisulphite solution (0.25% SO_2_), adjusted to pH 5.0 using lactic acid, and maintained under agitation (300 rpm) at 30 °C for 1.25 h in a laboratory fermenter (Biostat Bplus, Sartorius; Madrid, Spain) [18]. Following steeping, the grains were milled using a plate mill (Corona, Lambers & Cia; Bogotá, Colombia) in three passes of 1 min each. Germ separation was carried out by flotation in water, followed by rinsing with 1.0 L of ultrapure water to remove residual starch. The resulting slurry was passed through a 0.3 mm stainless steel sieve to recover the hulls, representing the QFi [10]. This material was then thoroughly washed and dried overnight at 40 °C in a forced-air oven. It was milled using an electric grinder (Aromatic, Taurus; Oliana, Spain), sieved to <425 µm, and finally stored in vacuum- sealed polyethylene bags at 4 °C until further analysis.

### 2.3. Pasta Production

Fresh pasta of tagliatelle shape (1 cm wide) was produced at laboratory scale using the automatic pasta maker Nina (Springlane GmbH; Düsseldorf, Germany). The process comprised three stages: 3 min of kneading, 6 min of resting, and 7 min of extrusion under controlled conditions. Seven formulations were prepared: a control batch consisting entirely of WF, alongside six fortified variants incorporating quinoa ingredients. For each variant, a 200 g dough mass was prepared, and the process was carried out twice. Whole quinoa flours from white (WQF), red (RQF), or black (BQF) quinoa replaced 25% of the WF in the fortified samples, while fibre-enriched pastas included 6–12% QFi obtained by wet milling from the same quinoa varieties; white (WQFi), red (RQFi), and black (BQFi) (Table 1). The substitution percentage for each QFi (12% WQFi, 7% RQFi, 6% BQFi) was calculated by determining the amount of fibre contributed by 25% WhQF in the fortified formulations and adjusting the added QFi so that both approaches provided an equivalent total dietary fibre content. Whole quinoa flours contained 12.4 g/100 g (white), 17.3 g/100 g (red), and 17.1 g/100 g (black) of TDF, whereas QFi obtained by wet milling were more concentrated, reaching 25.9 g/100 g (white), 61.9 g/100 g (red), and 70.4 g/100 g (black). Fresh egg yolk (16 g) was added to all formulations, with distilled water as the only additional liquid. Samples from each batch were allocated for cooking quality assessment, and the remainder was dried overnight at 40 °C in a forced-air oven, and then milled and stored in polyethylene bags at 4 °C for further analysis.

### 2.4. Proximate Composition of Fresh Pasta Samples

Moisture, starch, and TDF contents were assessed in accordance with the standardised analytical protocols outlined in AOAC Methods 925.09, 996.11, and 991.43, respectively [19]. Protein content was quantified via the Dumas combustion technique (N × 5.7), following the guidelines of ISO/TS 16634-2:2016 [20]. Lipid extraction was performed using the Randall extraction method (AOAC 2003.05—diethyl ether) with a SER 158/3 Solvent Auto Extractor (Velp Scientifica, Usmate, Italy) [21]. Ash content was measured following the AACC Official Method 08-03 [22]. All fresh pasta samples were dried at 40 °C, milled, and analysed in quadruplicate.

### 2.5. Cooking Properties

Amount of 25 g of fresh pasta samples were cooked (without salt) in 250 mL of distilled water until the optimal cooking time of the control was reached, determined by checking every 30 s and pressing a piece between the fingers to confirm the disappearance of the core. After cooking, samples were rinsed with distilled water and drained for 2 min [23]. For solid loss, 10 mL of the combined cooking and rinsing water was placed in preheated, pre-weighed porcelain crucibles and dried at 40 °C overnight [23]. Solid loss (%) was calculated according to Equation (1). Ash loss and water absorption were evaluated according to Alonso-Álvarez and Haros [24]. Ash loss (%) was then determined by incineration at 600 °C for 2 h in a muffle furnace (Nabertherm controller B170; Lilienthal, Germany) and obtained following Equation (2). Water absorption (%) was calculated as the weight difference between raw and cooked drained pasta, relative to the raw weight, as shown in Equation (3). The swelling factor was assessed as the ratio between cooked and uncooked weight, in accordance with Equation (4). All analyses were performed in quadruplicate.

(1) $$Solid\;loss\left(\%\right)=\frac{Ws}{Wuc}\times 100$$

(2) $$Ash\;loss\;\left(\%\right)=\frac{Wash}{Wuc}\times 100$$

(3) $$Water\;absorption\;\left(\%\right)=\frac{Wc-Wuc}{Wuc}\times 100$$

(4) $$Swelling\;factor=\frac{Wc}{Wuc}$$

where *W_s_* is the dry weight of solids recovered from cooking water (g); *W_uc_* is the weight of uncooked pasta (g); *W_ash_* is the weight of ash obtained after incineration (g); and *W_c_* is the weight of cooked and drained pasta (g).

### 2.6. Determination of Colour

The colour parameters of fresh and cooked pasta samples were assessed using a Chroma Meter CR 400 (Konica Minolta Sensing Inc.; Tokyo, Japan), previously calibrated with standard white reference plate (L* = 93.4, a* = 0.2, b* = 2.6). Measurements were performed using illuminant D65 and a 2° standard observer, operating in SCI (Specular Component Included) mode [25]. For each formulation, two independent batches were prepared, and three measurements were taken at different points of each sample surface. Cooked pasta samples were gently blotted with absorbent paper to remove excess surface moisture prior to measurement. Results were expressed in the CIELAB colour space, registering lightness (L*), red-green coordinate (a*), and yellow-blue coordinate (b*). In addition, chroma (C*) and hue angle (*h*°) were directly obtained from the instrument derived from the CIELAB coordinates. Chroma (C*) reflects colour saturation and *h*° defines its tone [26]. Colour differences (ΔE*) between samples groups were calculated using Equation (5). Furthermore, changes associated with cooking were assessed within each formulation (ΔE*c) using Equation (6), calculated as the difference between paired cooked (c) and uncooked (unc) tagliatelle.

The blackness index (*BI*) was obtained using Equation (7), representing the degree of darkness of the samples. Hunter’s Whiteness Index (*WI*) was calculated according to Equation (8), representing sample whiteness, as an indicator of their lightness and deviation from an ideal white surface [27].

(5) $$∆E^{*}=\sqrt{{∆L}^{*2}+{∆a}^{*2}+{∆b}^{*2}}$$

(6) $$∆E^{*}c=\sqrt{{(L}^{*}c-L^{*}unc{)}^{2}+{{(a}^{*}c-a^{*}unc)}^{2}+{{(b}^{*}c-b^{*}unc)}^{2}}$$

(7) $$\mathrm{BI}=100-L^{*}$$

(8) $$\mathrm{WI}=100-\sqrt{{\left(100-L^{*}\right)}^{2}+a^{*2}+b^{*2}}$$

The Just Noticeable Difference (JND) was interpreted from ∆E* values, where values below 1 are not perceptible, values between 1 and 3 are slightly perceptible, and values above 3 are clearly noticeable to human eye [28].

### 2.7. Determination of Phytate

Phytic acid or phytate (Ins*P*_6_) content in cooked pasta was quantified employing a commercial assay kit (K-Phyt 07/11, Megazyme, Bray, Ireland) and a spectrophotometer (SPECTROstar Nano, BMG LabTech; Ortenberg, Germany). Phytates were initially extracted from 1.0 g of the homogenised sample with 10 mL of 0.66 M hydrochloric acid. The extract subsequently underwent enzymatic dephosphorylation via phytase and alkaline phosphatase treatment, following the AOAC Method 986.11 [29]. Released phosphate was then determined by a colourimetric procedure using ammonium molybdate and ascorbic acid as chromogenic reagents. The intensity of the molybdenum blue complex formed, proportional to the amount of inorganic phosphate, was measured at 655 nm. Bound phosphorous, calculated as the difference between total and free phosphorus, was expressed as phytic acid using a gravimetric conversion factor. All analyses were performed in triplicate.

### 2.8. Determination of Minerals

Aliquots of 0.25 g from each cooked pasta formulation were subjected to acid digestion using 10 mL of 14 M nitric acid (Merck, Darmstadt, Germany) and 2 mL of hydrogen peroxide (30%, Panreac Química, Barcelona, Spain) in perfluoroalkoxy alkane (PFA) vessels. Digestion was performed using a microwave-assisted reaction system (MARS 6, One Touch Technology, CEM, Vertex; Madrid, Spain). The digestion programme consisted of a ramp of 25 min to reach 210 °C, followed by a holding time of 15 min, with microwave power automatically adjusted by the system to reach the programmed temperature. After digestion, the samples were transferred to plastic tubes and diluted to a final volume of 14 mL with ultrapure water. The concentrations of Ca, Fe, and Zn were subsequently determined by inductively coupled plasma mass spectrometry (ICP-MS; iCAP RQ, ThermoFisher Scientific; Waltham, MA, USA), as described by Sánchez et al. [30]. All measurements were conducted in quadruplicate.

### 2.9. Statistical Analysis

All statistical analyses were performed using R software (version 4.4.3). A two-way ANOVA was first conducted to evaluate the effects of phenotypic quinoa variety (white, red, black) and quinoa ingredient form (whole flour vs. fibre-rich fraction) on the compositional and technological variables, excluding the control formulation. When significant effects were detected, pairwise differences among quinoa-enriched samples were assessed using Tukey’s HSD test (*p* < 0.05). Subsequently, a one-way ANOVA, including all seven formulations, was performed for each response variable in order to obtain the global comparison across formulations. Tukey’s HSD test (*p* < 0.05) was again applied to this model to assign significance groupings. Pearson correlation coefficients were calculated to explore the relationships among compositional, technological, and colour parameters, and the resulting correlation matrix was visualised as a heatmap. In addition, a principal component analysis (PCA) was carried out to integrate the multivariate structure of the dataset. Both score and loading plots were examined to interpret sample clustering and variable contributions to the principal components.

## 3. Results and Discussion

### 3.1. Fresh Pasta Composition

The chemical composition of the fresh pasta formulations is presented in Table 2. Moisture content was significantly higher in the control pasta and in samples supplemented with the QFi, compared to those fortified with WhQF. This behaviour could be attributed to differences in water addition during processing and to higher water-binding capacity of the QFi, which required increased water incorporation to achieve dough consistency for extrusion. Although starch content did not differ significantly between the control and fortified pastas, a slight decrease was observed in all quinoa-substituted (WhQF or QFi) formulations. Protein levels were maintained across all formulations, showing no significant differences when compared to the control. This suggests that both substitution approaches effectively preserve the overall protein contribution in the final product. Additionally, lipid and ash contents were significantly higher in pastas enriched with WhQFs, partly due to the intrinsic composition of these flours. With the exception of protein, the levels of minerals, lipids, and starch decreased in the formulations containing the QFi; this fraction is largely devoid of these nutrients compared to the formulations enriched with WhQF, as expected. A similar trend was reported previously in bread fortified with quinoa flour and fibre [5]. Pasta samples with WhQFs revealed significantly higher total dietary fibre (TDF) than the control, with even greater rises in those enriched with QFi, particularly the BQFi formulation. Adding small amounts of QFi boosted the TDF of fresh pasta by over 50% compared to the control and by 17–45% relative to the corresponding WhQF. Consequently, a 100 g intake of fresh pasta enriched with QFi would contribute more substantially to the recommended adequate daily intake of dietary fibre, set at 25 g by the WHO; this intake exceeds that provided by both the control pasta and those containing WhQF. According to Regulation (EU) No. 1924/2006 [31], all formulations qualify as “source of fibre” (≥3 g/100 g), while BQF pasta and those containing QFi meet the criteria for a “high fibre” claim (≥6 g/100 g).

### 3.2. Effect of Quinoa Flour and Quinoa Fibres on Technological Parameters of Cooked Pasta

Table 3 presents the effect of incorporating WhQF and its QFi on the cooking properties of wheat tagliatelle. The differences observed in water absorption during cooking appear to reflect not only the hydration properties of the added quinoa ingredients but also their interaction with the wheat-based matrix. Pastas enriched with WhQF showed significantly higher water absorption than the control, mostly in samples containing WQF and BQF. This increment can be attributed in part to the higher TDF and protein content of WhQF, which are known to augment water-binding capacity and modify the formation and continuity of the gluten network by diluting gluten proteins and competing for water during dough hydration [32]. Dietary fibre components, particularly insoluble fractions, may interfere with gluten development and promote a more heterogeneous dough structure, facilitating water penetration during cooking [33,34]. Moreover, the slight reduction in starch content observed in the enriched samples and the relatively coarse particle size of WhQF and QFi could lead to a more open matrix structure, facilitating greater water penetration and swelling, while simultaneously causing partial disruption of the gluten network [33]. In line with this, the swelling factor demonstrated an uplift in all fortified samples compared to the control, with the highest values recorded for WQFi, WQF, and BQF. Similarly, pastas fortified with QFi also resulted in elevated levels of water absorption, particularly the sample containing WQFi. Although a previous study had identified this RQFi as having the highest water-holding capacity (WHC) and swelling capacity (SC) [10], this was not fully reflected in the cooked pasta. Instead, the formulation with WQFi retained the most water, suggesting that the hydration behaviour of fibres under cooking conditions is influenced by more than their isolated WHC and SC values. Indeed, fibre functionality in composite dough systems depends on several factors, including particle size, porosity, and interactions with gluten and starch components within the matrix, which determine water accessibility and swelling behaviour during thermal processing [35,36]. Overall, both WhQFs and QFi improved the water absorption capacity of pasta. Regarding mineral loss, all fortified samples exhibited ash values in a similar range to the control, with the pasta containing BQFi showing the lowest ash loss. This may reflect a more stable mineral matrix or stronger retention or inorganic components during cooking, potentially linked to the high insoluble fibre and matrix compactness of this formulation [24]. Such effects have been attributed to stronger protein–starch networks and mineral–fibre interactions, which help reduce leaching during cooking [37]. This observation aligned with the two-way ANOVA, which revealed significant effects of quinoa form (WhQFs and QFi), colour variety (white, red and black), and their interaction on ash loss, indicating that mineral retention during cooking could be influenced by both the type of quinoa ingredient and the variety used. Solid loss reflects the integrity and cohesion of the gluten–starch matrix. As expected, the control pasta had the lowest values, consistent with its relatively more continuous protein network. In contrast, pastas substituted with WhQFs, especially WQF, suffered significantly higher losses. This behaviour is likely associated with the dilution and partial disruption of the gluten structure caused by the incorporation of coarse, non-gluten particles, together with the leaching of soluble compounds [37,38]. Similar effects have been widely reported in fibre-fortified pasta, where reduced gluten continuity weakens the matrix and facilitates the release of starch and soluble materials during cooking [39]. Interestingly, formulations enriched with QFi were found to have intermediate values of solid loss. This may be partly explained by the lower inclusion level of the QFi (6–12%) compared to the WhQFs (25%), which likely resulted in less disruption of the gluten–starch matrix and a reduced number of leachable compounds. Additionally, the water-binding properties of dietary fibre could have contributed to maintaining dough cohesion, further limiting the migration of solids into the cooking water [40,41].

### 3.3. Colour

**Figure 1:**
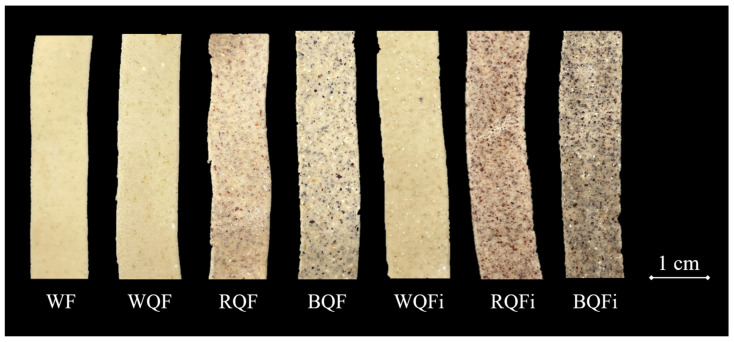
Fresh wheat flour-based pasta samples. Abbreviations: WF (wheat flour-control); and wheat flour-based pastas added with: WQF (White quinoa flour); RQF (Red quinoa flour); BQF (Black quinoa flour); WQFi (White quinoa fibre); RQFi (Red quinoa fibre); BQFi (Black quinoa fibre).

**Figure 2:**
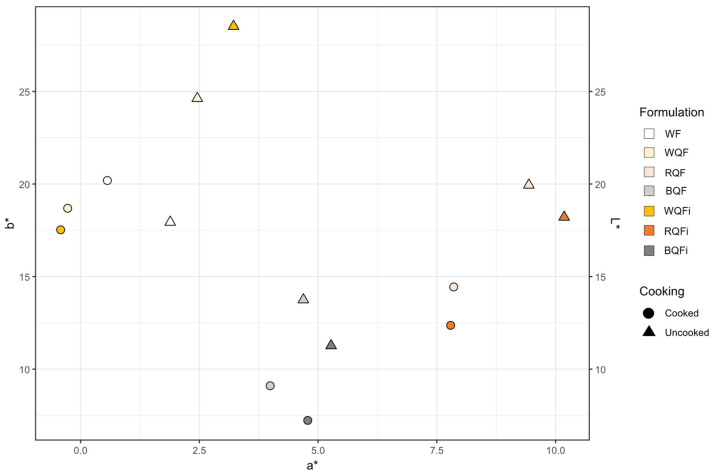
Chromatic distribution of cooked and uncooked wheat flour-based pastas (b* vs. a*) with L* values on a secondary axis. Abbreviations: WF (wheat flour-control); and wheat flour-based pastas added with: WQF (white quinoa flour); RQF (red quinoa flour); BQF (black quinoa flour); WQFi (white quinoa fibre); RQFi (red quinoa fibre); and BQFi (black quinoa fibre).

Colour is a key quality parameter in pasta products, strongly influencing consumer acceptance. The chromatic values of the WF used to make the tagliatelle were: L*: 90 ± 0.01; a*: −0.97 ± 0.02; b*: 8.67 ± 0.02; C*: 8.73 ± 0.01; *h*°: 96.4 ± 0.1. Table 4 presents the colour attributes of uncooked and cooked pasta formulations. The highest lightness (L*) value was observed in the control, whereas pasta containing BQFi, both in uncooked and cooked states, exhibited the lowest L*, indicating a markedly darker appearance (Figure 1). Substitution with RQF and BQF, as well as their QFi, also supported redness (a*) and, reversely, yellowness (b*), reflecting the visual contribution of natural pigments in these varieties, in line with previous reports on phenolic compounds in quinoa [7,42]. Similar trends involving reduced L* and greater a* and b* values in uncooked pasta were also reported by Torres et al. [43] when incorporating quinoa flour into extruded wheat pasta. Cooking led to a general decrease in a* values across most samples, likely due to pigment degradation by thermal processing, leaching of water-soluble phenolics, and matrix hydration effects [7,44,45], with a particularly notable drop in wheat pastas formulated with BQF and RQF. In contrast, WQF and WQFi contributed to higher yellowness values, suggesting a brighter yellow tone compared to pastas formulated with pigmented varieties, as expected. Overall, incorporation of red and black quinoa varieties led to darker, redder, and less yellow pastas. Figure 2 shows the chromatic distribution of the samples (b* versus a*) together with the L* values on the secondary axis. In terms of total colour difference (ΔE*), most formulations revealed values above 3, indicating a clearly perceptible difference to the human eye according to the JND criterion [28]. The only exception was cooked pasta with WQF, which had a ΔE* value of 2.4, corresponding to a slightly perceptible difference. Conversely, all samples presented ΔE*c values above 3, confirming that cooking itself induced noticeable colour changes irrespective of formulation. Chroma (C*) varied depending on the type of quinoa ingredient incorporated. Pasta containing RQF generally yielded higher C* values, indicating more vivid colours, while wheat-BQF and wheat-BQFi pastas showed lower C*, suggesting a duller and less saturated appearance. Variations in *h*° also indicated shifts in colour tone, with pigmented quinoa containing formulations tending towards redder hues, whereas wheat-WQF pasta maintained a more yellowish tone. These trends are supported by *BI* and *WI* values, where quinoa-enriched samples, particularly those containing RQFi and BQFi, displayed higher *BI* and lower *WI* values, confirming their darker and less bright appearance. Despite these visual effects, the nutritional enhancements provided by quinoa, such as higher dietary fibre and mineral content, may offset potential drawbacks in consumer acceptance [1,46].

### 3.4. Mineral Content and Recommended Daily Allowances (RDAs)/Population Reference Intakes (PRIs)

**Figure 3:**
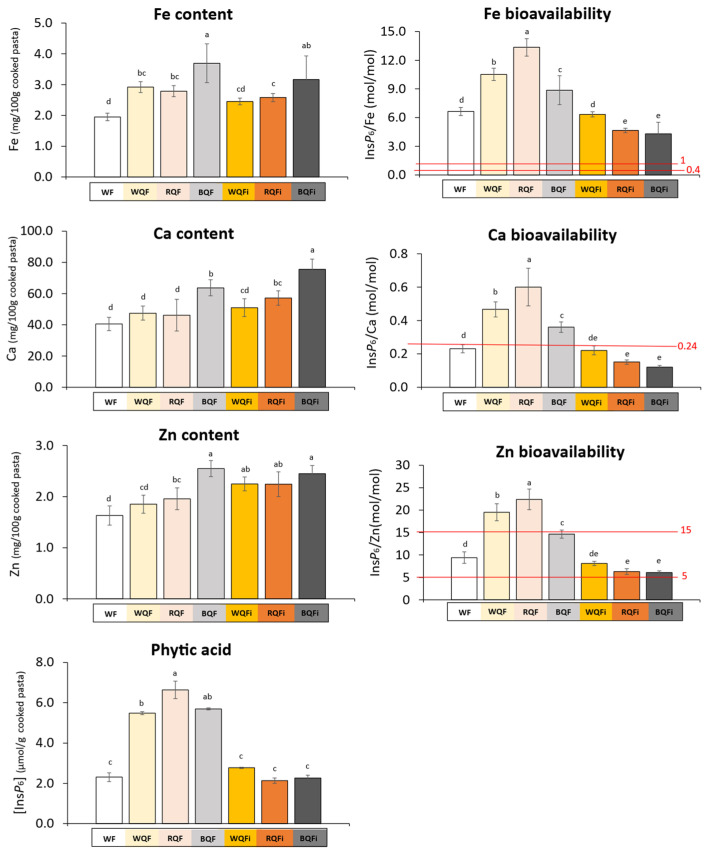
Phytic acid and mineral bioavailability estimation in cooked pasta. Threshold rations (Ins*P*_6_/mineral) for mineral availability inhibition (marked with the red lines) [53,55,56,58,59]. Values are expressed as mean ± standard deviation (*n* ≥ 3) on a dry matter of cooked pasta. The bars of each parameter followed by the same letter do not show significant differences at a 95% confidence level. Abbreviations: WF (wheat flour-control); and wheat flour-based pastas added with: WQF (white quinoa flour); RQF (red quinoa flour); BQF (black quinoa flour); WQFi (white quinoa fibre); RQFi (red quinoa fibre); and BQFi (black quinoa fibre).

The partial substitution of WF with WhQFs and QFi significantly raised the levels of Fe, Ca, and Zn in the cooked pasta samples compared with the control formulation (*p* < 0.05); (Figure 3). The WF used in pasta production contained (on a dry matter basis): Ca, 26.4 mg/100 g; Fe, 1.24 mg/100 g; and Zn, 1.59 mg/100 g (all expressed per 100 g of cooked pasta). Among the enriched formulations, those incorporating BQF or BQFi demonstrated the greatest mineral enrichment. This outcome is consistent with the intrinsically high Ca and Fe concentrations of these raw materials (BQF: 71 mg Ca/100 g, 6.3 mg Fe/100 g; BQFi: 82 mg Ca/100 g, 7.6 mg Fe/100 g; all values expressed per 100 g of cooked pasta), in which these minerals become concentrated within the QFi obtained through the wet milling process [10]. By contrast, Zn content produced a more moderate increase, with the highest values in pasta containing BQF, followed by BQFi pasta, both significantly higher than the control and the WQF and RQF formulations. This pattern reflects the lower Zn contribution of the QFi, particularly from RQFi and BQFi [10], and aligns with observations in wheat and buckwheat, where Zn is predominantly localised in the embryo and aleurone layer rather than in the fibre fraction [47,48]. Interestingly, although the inclusion level of QFi was lower than that of WhQFs the high concentration of Ca and Fe in these ingredients translated into notable rise in the final mineral content of the enriched pastas. In several cases, the mineral content in QFi-enriched formulations approached or even surpassed that of the WhQF-enriched ones. This highlights the efficiency of QFi in delivering essential micronutrients, even when included at relatively low substitution rates. These trends were supported by the two-way ANOVA, which showed significant main effects of quinoa form (WhQs and QFi) and quinoa variety (white, red, and black) for Fe and Ca, whereas Zn additionally exhibited a significant interaction between both factors, indicating that the influence of quinoa form on Zn content depended on the quinoa variety used.

The mineral contribution from a serving of 100 g of cooked pasta was evaluated in relation to the adult Recommended Daily Allowances (RDAs) established by the Food and Agriculture Organization (FAO) [49] and the Population Reference Intakes (PRIs) defined by the European Food Safety Authority (EFSA) [50,51,52], if no mineral absorption inhibitors are present (Table 5). In populations following partial- or meat-free diets, Fe bioavailability is considerably reduced, due to the absence of haem iron and the higher intake of absorption inhibitors such as phytates found in legumes, cereals, oilseeds, and nuts, among others [49,53]. Consequently, RDAs for Fe are adjusted based on the presumed bioavailability associated with different dietary patterns. Zn bioavailability is similarly influenced by dietary components, particularly the presence of phytates [54]. The FAO classifies Zn bioavailability into three levels based on the molar ratio of phytic acid (Ins*P*_6_) to Zn: high (<5), moderate (5–15), and low (>15), with ratios above 15 known to markedly reduce Zn absorption and even produce a negative balance [49,55]. These categories broadly correspond to estimated absorption levels of about 50%, 30%, and 15%, respectively [56]. In contrast, the EFSA defines four categories according to daily Ins*P*_6_ intake—300, 600, 900, and 1200 mg—to estimate mineral bioavailability in various dietary contexts [52]. Ca contribution to RDAs/PRIs was observed to be higher in all quinoa-enriched pasta samples compared with the control. Among the studied formulations, those containing BQF or BQFi delivered the highest Ca contributions.

Overall, the assessment of mineral contribution per serving reinforces the compositional trends while providing nutritional information relevant to dietary intake. All quinoa-enriched formulations delivered higher Ca, Fe, and Zn contributions than the control, although the extent of improvement varied with both quinoa variety and ingredient type. Black quinoa flour and its fibre-rich fraction provided the greatest improvements, reflecting their intrinsically elevated mineral concentrations. Importantly, the QFi, despite being incorporated at lower levels than the WhQFs, often achieved comparable contributions, confirming their capacity to act as concentrated mineral sources and indicating their suitability for targeted fortification strategies. Collectively, these findings could demonstrate that the strategic selection of quinoa ingredients, particularly those derived from black varieties, enables the development of food products with meaningfully enhanced mineral density.

### 3.5. Phytate Impact on Mineral Absorption

Phytic acid or phytate (Ins*P*_6_) is a well-known chelator of divalent minerals such as Ca, Fe, and Zn. By forming insoluble complexes in the gastrointestinal tract, it can markedly reduce mineral bioavailability. QFi obtained through wet milling process was found to have a notably reduced phytic acid content, explained by phytate leaching during the steeping step, which may suggest improved mineral absorption potential in formulations enriched with these raw materials. To evaluate this potential, molar ratios of phytic acid to minerals (Ins*P*_6_/Ca, Ins*P*_6_/Fe, Ins*P*_6_/Zn) were calculated, as these are widely recognised predictive indicators of mineral bioavailability [57]. The phytate content of the pasta formulations and the threshold values for phytate-to-mineral molar ratios are presented in Figure 3. The WF used as a raw material for all pasta formulations contained 3.18 µmol/g of phytates on a dry matter basis. As expected, pastas enriched with WhQFs possessed higher phytic acid levels, with the greatest concentration found in the formulation containing RQF, consistent with the high phytate content observed in this particular raw material [10]. In contrast, formulations incorporating QFi contained lower phytic acid concentrations, with values comparable to those of the control pasta. According to the two-way ANOVA, phytic acid levels in the tagliatelle were primarily determined by the ingredient form (WhQFs and QFi) rather than by the phenotypic quinoa variety. However, the significant interaction between both factors revealed that the magnitude of phytate reduction in QFi-enriched pastas differed among varieties. Considering the relatively low phytate content observed, the potential implications for mineral bioavailability can be assessed using established phytate-mineral molar ratio thresholds [57]. Human studies demonstrated that an Ins*P*_6_/Ca ratio exceeding 0.24 is sufficient to impair Ca absorption [58]. Similarly, the inhibitory effect of phytic acid on Fe uptake diminish markedly when the Ins*P*_6_/Fe molar ratio falls below 1.0 [53], and optimal absorption is generally associated with ratios below 0.4 [59]. Zn absorption is particularly sensitive to the presence of phytic acid [54], and the inhibitory effect increases sharply as the Ins*P*_6_/Zn molar ratio rises. Ratios below 5 are generally associated with high Zn bioavailability, around 50% ingested Zn. Values between 5 and 15 correspond to moderate bioavailability, with absorption decreasing to approximately 30%. When the ratio exceeds 15, Zn bioavailability becomes markedly low, often limited to 15% [55,56]. Among the samples analysed, only the pasta formulations containing WhQFs exceeded the 0.24 threshold ratio for Ins*P*_6_/Ca, suggesting a potential reduction in Ca bioavailability for these products (Figure 3). Fe absorption appeared to be affected in all formulations, though to a lesser extent in both the control pasta and those enriched with QFi (Figure 3). Regarding Zn, most formulations showed Ins*P*_6_/Zn molar ratios between 5 and 1, indicating moderate Zn bioavailability. However, WQF and RQF formulations exhibited ratios above 15, suggesting markedly reduced Zn bioavailability. Among these, the pasta added with RQF displayed the highest ratio, likely due to its higher phytate content, indicating a greater limitation for Zn absorption (Figure 3). These results could indicate that substitution with QFi, rather than WhQFs, may favour mineral absorption by minimising phytate inhibition.

### 3.6. Multivariate Analysis (Correlation and PCA)

**Figure 4:**
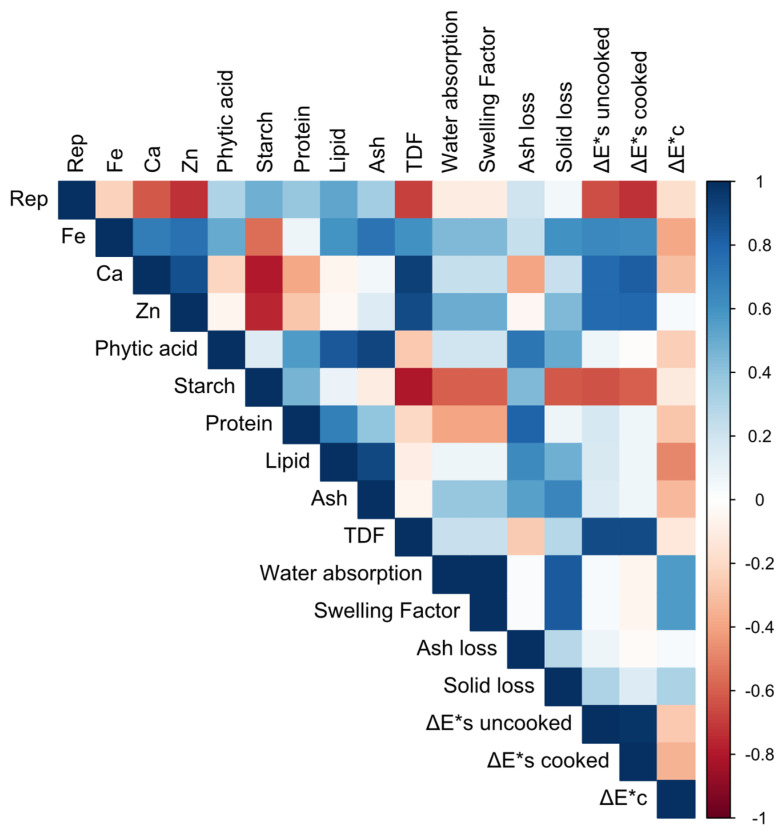
Pearson correlation matrix of measured parameters. Colours represent correlation coefficients from −1 (red, negative correlation) to +1 (blue, positive correlation), with values near 0 indicating weak or no linear relationship.

Figure 4 depicts the Pearson correlation matrix, providing an overview of the interrelationships among compositional and technological parameters across the studied formulations. Strong positive correlations were observed among mineral contents (Fe, Ca, and Zn), indicating that these elements tend to co-vary across formulations, likely reflecting their common origin in the raw materials. Protein, lipid, and ash contents were also positively associated, suggesting that formulations richer in these components share a similar compositional profile. In contrast, starch content showed negative correlations with protein, lipids, ash, and TDF, highlighting an inverse relationship between carbohydrate content and other nutritional components.

Phytic acid displayed moderate positive correlations with minerals and ash, aligning with its known affinity for binding divalent cations, while showing weaker associations with functional properties. Modest associations were observed between compositional variables and technological properties, such as water absorption, swelling factor, and cooking losses, suggesting that the macronutrient profile may influence the technological behaviour of the samples.

**Figure 5:**
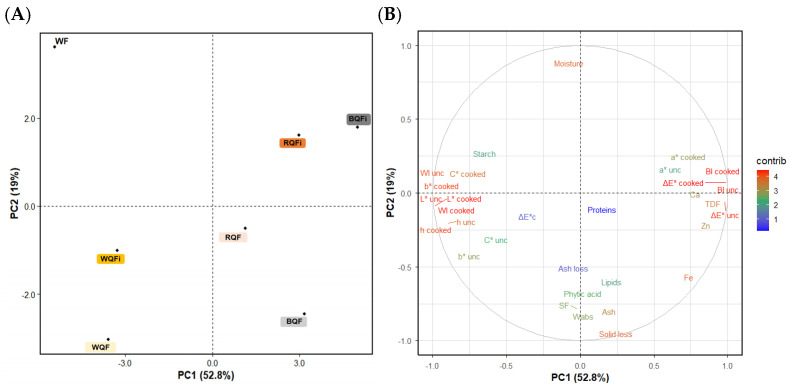
Principal component analysis (PCA) score plot (**A**) and variable loading plot (**B**) for PC1 vs. PC2. Variable contributions to PC1 and PC2 are shown using a blue-to-red colour scale (low to high). Abbreviations: WF (wheat flour-control); and wheat flour-based pastas added with: WQF (white quinoa flour); RQF (red quinoa flour); BQF (black quinoa flour); WQFi (white quinoa fibre); RQFi (red quinoa fibre); and BQFi (black quinoa fibre).

A principal component analysis (PCA) was performed to integrate the compositional and technological parameters of the studied formulations. The first two components explained 71% of the total variance (PC1 = 52.8% and PC2 = 19%), providing a robust representation of the multivariate structure. The score plot (Figure 5A) revealed a clear separation between control pasta and all quinoa-enriched formulations, confirming the incorporation of WhQFs and QFi substantially modified the overall product profile. Along PC1, the control sample was positioned opposite to all fortified variants, driven by its higher starch content and lower levels of protein, minerals, lipids, ash, and TDF. Formulations containing WhQF and QFi shifted in the same direction, indicating that even the lower inclusion levels of QFi were sufficient to generate a compositional signature comparable to that produced by replacing 25% of WF with WhQF. This supports the effectiveness of the wet milling isolates as concentrated sources of fibre and nutrients. PC2 captured variability mainly associated with colour attributes, which differentiated formulations according to quinoa types (white, red, and black). Samples added with red and black quinoa ingredients (RQF, BQF, RQFi, and BQFi) tended to separate from those enriched with WQF or WQFi, reflecting the influence of pigmented varieties on chromatic parameters. Consistent with the loading distribution (Figure 5B), colour parameters dominated PC2, whereas phytic acid indicate a weak representation in the PC1–PC2 plane despite its moderate overall contribution to the PCA. This behaviour reflects that phytic acid varied primarily with the quinoa ingredient form (WhQF vs. QFi) rather than with phenotypic variety, as confirmed by the two-way ANOVA. Overall, the PCA demonstrates that quinoa enrichment, regardless of whether it is introduced as WhQFs or QFi, drives a clear shift in the nutritional profile of the wheat flour-based tagliatelle.

## 4. Conclusions

Wet milled quinoa fibre-rich fraction can be effectively used to enrich the nutritional profile of pasta products. Their incorporation enabled a substantial increase in dietary fibre without compromising the cooking behaviour of the tagliatelle. In addition, QFi concentrates relevant minerals and provides natural pigments with antioxidant potential, contributing to the functional value of the final product. Importantly, its low phytic acid content compared with WhQFs suggests a more favourable scenario for mineral bioavailability. Overall, wet milled QFi could represent a valuable strategy for improving the nutritional quality of cereal-based foods.

## Figures and Tables

**Table 1:** Wheat flour-based pasta formulations with whole quinoa flours and fibre-rich fraction.

Ingredients	Units	Wheat Flour-Based Pasta Formulations						
		WF	WQF	RQF	BQF	WQFi	RQFi	BQFi
Wheat flour	g	200	150	150	150	177	186	188
Quinoa ingredient	g	-	50 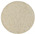	50 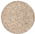	50 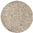	23 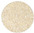	14 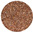	12 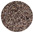
Fresh egg yolk	g	16	16	16	16	16	16	16
Distilled water	mL	62	62	62	62	78	80	80
Substitution rate	% (in wheat flour basis)	-	25	25	25	12	7	6

Abbreviations: WF (wheat flour); WQF (white quinoa flour); RQF (red quinoa flour); BQF (black quinoa flour); WQFi (white quinoa fibre); RQFi (red quinoa fibre); BQFi (black quinoa fibre).

**Table 2:** Effect of quinoa flour and quinoa fibre-rich fraction on chemical composition of fresh pasta ^1,2^.

Parameter	Units ^2^	Wheat Flour-Based Pasta Formulations						
		WF	WQF	RQF	BQF	WQFi	RQFi	BQFi
Moisture ^3^	g/100 g	37.6 ± 0.8 a	31.4 ± 0.8 c	32.8 ± 0.6 bc	32.2 ± 0.2 bc	35.3 ± 0.7 ab	36.9 ± 0.3 a	36.2 ± 0.5 a
Starch	g/100 g	74.7 ± 1.9 a	72.1 ± 4.3 a	73.1 ± 2.1 a	72.3 ± 3.5 a	71.7 ± 8.5 a	72.0 ± 2.7 a	70.6 ± 2.1 a
Proteins	g/100 g	12.3 ± 0.4 a	12.4 ± 0.3 a	12.8 ± 0.2 a	12.5 ± 0.1 a	11.8 ± 0.4 a	12.7 ± 0.1 a	11.8 ± 0.6 a
Lipids	g/100 g	3.1 ± 0.3 c	4.2 ± 0.2 a	3.9 ± 0.3 ab	4.1 ± 0.1 a	2.7 ± 0.3 c	3.4 ± 0.2 bc	3.1 ± 0.1 c
Ash	g/100 g	0.769 ± 0.083 b	1.097 ± 0.014 a	1.025 ± 0.003 a	1.102 ± 0.045 a	0.824 ± 0.003 b	0.790 ± 0.025 b	0.867 ± 0.008 b
TDF	g/100 g	4.3 ± 0.3 e	5.6 ± 0.5 de	5.8 ± 0.2 b	7.8 ± 0.4 abc	6.4 ± 0.2 bcd	8.1 ± 0.4 ab	9.1 ± 0.3 a

^1^ Values are expressed as mean ± standard deviation (*n* = 4). Values followed by the same letter in the same line are not significantly different at a 95% confidence level (Tukey’s test). ^2^ Dry matter, d.m. ^3^ Wet basis. Abbreviations: TDF = Total Dietary Fibre; WF (wheat flour-control); WQF (White quinoa flour); RQF (Red quinoa flour); BQF (Black quinoa flour); WQFi (White quinoa fibre); RQFi (Red quinoa fibre); BQFi (Black quinoa fibre). Fibre-rich fraction (QFi) was incorporated at specifically adjusted substitution levels (12% WQFi, 7% RQFi, and 6% BQFi) to ensure a dietary fibre content equivalent to that of pasta formulated with 25% whole quinoa flour (WhQF). Abbreviations: WF (wheat flour-control); and wheat flour-based pastas added with: WQF (White quinoa flour); RQF (Red quinoa flour); BQF (Black quinoa flour); WQFi (White quinoa fibre); RQFi (Red quinoa fibre); BQFi (Black quinoa fibre).

**Table 3:** Effect of quinoa flour and quinoa fibre-rich fraction on technological parameters of cooked pasta ^1,2^.

Parameter	Units	Wheat Flour-Based Pasta Formulations						
		WF	WQF	RQF	BQF	WQFi	RQFi	BQFi
***Cooking properties***								
Water absorption ^3,4^	%	82.19 ± 0.36 d	96.20 ± 0.0 2 ab	86.92 ± 0.81 cd	96.16 ± 0.27 ab	100.86 ± 2.48 a	88.44 ± 0.39 c	90.18 ± 2.30 bc
Swelling factor	-	1.8219 ± 0.0036 d	1.9620 ± 0.0002 ab	1.8692 ± 0.0081 cd	1.9616 ± 0.0027 ab	2.0086 ± 0.0248 a	1.8844 ± 0.0039 c	1.9018 ± 0.0230 bc
Ash loss	%	0.0140 ± 0.0024 b	0.0149 ± 0.0004 ab	0.0186 ± 0.0028 a	0.0192 ± 0.0015 a	0.0136 ± 0.0006 bc	0.0158 ± 0.0011 ab	0.0090 ± 0.0025 c
Solid loss	%	0.23 ± 0.03 b	0.35 ± 0.02 a	0.30 ± 0.02 ab	0.32 ± 0.02 a	0.32 ± 0.02 a	0.30 ± 0.07 ab	0.29 ± 0.05 ab

^1^ Values are expressed as mean ± standard deviation (*n* ≥ 3). Values followed by the same letter in the same line are not significantly different at a 95% confidence level (Tukey’s test). ^2^ Dry matter, d.m. ^3^ Wet basis. ^4^ Cooking time: 5.0 min. Abbreviations: TDF = Total Dietary Fibre; WF (wheat flour-control); and wheat flour-based pastas added with: WQF (white quinoa flour); RQF (red quinoa flour); BQF (black quinoa flour); WQFi (white quinoa fibre); RQFi (red quinoa fibre); and BQFi (black quinoa fibre). Fibre-rich fraction (QFi) was incorporated at specifically adjusted substitution levels (12% WQFi, 7% RQFi, and 6% BQFi) to ensure a dietary fibre content equivalent to that of pasta formulated with 25% whole quinoa flour (WhQF).

**Table 4:** Colour parameters of wheat flour-based pasta formulations ^1^.

Parameter ^2^	Cooking	Wheat Flour-Based Pasta Formulations						
		WF	WQF	RQF	BQF	WQFi	RQFi	BQFi
L*	Uncooked	81.66 ± 1.79 a	73.87 ± 0.52 b	55.40 ± 3.44 de	51.74 ± 1.52 ef	74.24 ± 0.10 b	47.48 ± 0.14 fg	46.87 ± 0.01 fg
	Cooked	76.4 ± 1.4 ab	74.9 ± 1.0 b	57.3 ± 0.9 d	54.4 ± 2.0 de	68.8 ± 0.2 c	52.5 ± 1.7 e	46.7 ± 1.1 g
a*	Uncooked	1.89 ± 0.09 g	2.46 ± 0.12 fg	9.44 ± 0.71 a	4.69 ± 0.19 cd	3.22 ± 0.12 ef	10.18 ± 0.01 a	5.27 ± 0.01 c
	Cooked	0.56 ± 0.03 h	−0.27 ± 0.14 h	7.86 ± 0.36 b	3.99 ± 0.26 de	−0.42 ± 0.02 h	7.79 ± 0.55 b	4.78 ± 0.22 c
b*	Uncooked	17.9 ± 0.2 cd	24.6 ± 0.3 b	19.9 ± 1.6 c	13.7 ± 0.5 ef	28.5 ± 0.4 a	18.2 ± 0.1 cd	11.3 ± 0.1 fg
	Cooked	20.2 ± 0.5 c	18.7 ± 0.6 cd	14.4 ± 0.9 e	9.1 ± 0.9 gh	17.5 ± 0.4 d	12.4 ± 1.2 f	7.2 ± 0.8 h
C*	Uncooked	18.0 ± 0.2 def	24.7 ± 0.3 b	22.1 ± 1.8 c	14.5 ± 0.5 gh	28.8 ± 0.4 a	20.9 ± 0.1 cd	12.4 ± 0.1 h
	Cooked	20.2 ± 0.5 cde	18.7 ± 0.6 de	16.4 ± 0.9 fg	9.9 ± 0.9 i	17.5 ± 0.4 ef	14.6 ± 1.3 gh	8.7 ± 0.7 i
*h*°	Uncooked	84.0 ± 0.3 c	84.3 ± 0.2 bc	64.7 ± 0.2 e	71.2 ± 0.7 d	83.6 ± 0.1 c	60.8 ± 0.2 fg	64.9 ± 0.2 ef
	Cooked	88.4 ± 0.1 ab	90.2 ± 1.1 a	61.4 ± 0.7 f	66.2 ± 1.7 e	91.4 ± 0.1 a	57.6 ± 2.0 gh	56.4 ± 2.7 h
ΔE*	Uncooked	0.00 h	10.30 ± 0.23 fg	27.51 ± 2.99 cd	30.35 ± 1.53 bc	12.99 ± 0.27 f	35.17 ± 0.14 a	35.59 ± 0.02 a
	Cooked	0.00 h	2.4 ± 0.9 h	21.2 ± 0.9 e	24.8 ± 2.1 d	8.1 ± 0.1 g	26.2 ± 1.8 d	32.7 ± 1.3 ab
ΔE*c	-	6.0 ± 1.1 cd	6.7 ± 0.4 c	6.1 ± 1.0 c	5.8 ± 0.5 c	12.8 ± 0.3 a	8.3 ± 0.8 b	4.3 ± 0.6 d
*BI*	Uncooked	18.34 ± 1.79 g	26.13 ± 0.52 f	44.61 ± 3.44 cd	48.26 ± 1.52 bc	26.76 ± 0.10 f	52.52 ± 0.14 ab	53.13 ± 0.01 ab
	Cooked	23.6 ± 1.4 fg	25.1 ± 1.0 f	42.7 ± 0.9 d	45.6 ± 2.0 cd	31.2 ± 0.2 e	47.5 ± 1.7 c	53.4 ± 1.1 a
*WI*	Uncooked	74.26 ± 1.42 a	64.01 ± 0.20 c	50.14 ± 2.34 e	49.60 ± 1.35 e	61.44 ± 0.24 c	43.49 ± 0.10 f	45.43 ± 0.04 f
	Cooked	68.9 ± 0.8 b	68.7 ± 0.5 b	54.2 ± 1.0 d	53.4 ± 1.8 d	64.2 ± 0.3 c	50.3 ± 1.4 e	45.9 ± 1.0 f

Values are expressed as mean ± standard deviation (*n* ≥ 3). ^1^ Fibre-rich fraction (QFi) was incorporated at specifically adjusted substitution levels (12% WQFi, 7% RQFi, and 6% BQFi) to ensure a dietary fibre content equivalent to that of pasta formulated with 25% whole quinoa flour (WhQF). Values followed by different letters indicate significant differences among the combinations of formulation type and cooking condition (Tukey’s test, 95% confidence level). For ΔE*c, letters are based on a one-way ANOVA across formulations. ^2^ Colour parameters: L* (lightness), a* (redness-greenness) and b* (yellowness-blueness), C* (chroma); *h*° (hue angle) values; ΔE* (difference between the sample and the control sample; Equation (5)); ΔE*c (difference between cooked and uncooked tagliatelle from same formulation; Equation (6)); *BI* (Blackness Index; Equation (7)); *WI* (Hunter’s Whiteness Index; Equation (8)). Abbreviations: WF (wheat flour-control); and wheat flour-based pastas added with: WQF (white quinoa flour); RQF (red quinoa flour); BQF (black quinoa flour); WQFi (white quinoa fibre); RQFi (red quinoa fibre); and BQFi (black quinoa fibre).

**Table 5:** Theoretical contribution to dietary reference intakes for minerals and fibre of wheat flour-based cooked pasta formulations ^1^.

Parameter		Units ^2^	Wheat Flour-Based Pasta Formulations						
			WF	WQF	RQF	BQF	WQFi	RQFi	BQFi
**% RDA or PRI contribution ^2^** (male/female)		**mg/day**							
Ca	FAO	1000/1000	4/4	5/5	5/5	6/6	5/5	6/6	8/8
	EFSA	950/950	4/4	5/5	5/5	7/7	5/5	6/6	8/8
Fe	FAO_5_	27.4/58.8	7/3	11/5	10/5	13/6	9/4	9/4	12/5
	FAO_10_	13.7/29.4	14/7	21/10	20/9	27/13	18/8	19/9	23/11
	FAO_12_	11.4/24.5	17/8	26/12	24/11	32/15	21/10	23/11	28/13
	FAO_15_	9.1/19.6	21/10	32/15	31/14	41/19	27/13	28/13	35/16
	EFSA	11/16	18/12	27/18	25/17	34/23	22/15	23/16	29/20
Zn	FAO_high_	4.2/3	39/54	44/62	47/65	61/85	54/75	53/75	58/82
	FAO_moderate_	7/4.9	23/33	26/38	28/40	36//52	32/46	32/46	35/50
	FAO_low_	14/9.8	12/17	13/19	14/20	18/26	16/23	16/23	17/25
	EFSA_300_	9.4/7.5	17/22	20/25	21/26	27/34	24/30	24/30	26/33
	EFSA_600_	11.7/9.3	14/18	16/20	17/21	22/27	19/24	19/24	21/26
	EFSA_900_	14.0/11.0	12/15	13/17	14/18	18/23	16/20	16/20	17/22
	EFSA_1200_	16.3/12.7	10/13	11/15	12/15	16/20	14/18	14/18	15/19
**% Adequate fibre intake contribution ^3^**			16	20	21	28	23	31	33

^1^ Fibre-rich fraction (QFi) was incorporated at specifically adjusted substitution levels (12% WQFi, 7% RQFi, and 6% BQFi) to ensure a dietary fibre content equivalent to that of pasta formulated with 25% whole quinoa flours (WhQFs), respectively. ^2^ FAO (Food and Agriculture Organization), RDAs (Recommended Daily Allowances), EFSA (European Food Safety Authority), PRIs (Population Reference Intakes) contribution (%) for a daily average intake of 100 g of fresh pasta if mineral absorption inhibitors are absent. RDAs/PRIs in mg/day for adults (males/females) ≥ 18 years [49,50,51,52]. The FAO/RDAs for Fe are based on the type of the diet, considering different levels of Fe bioavailability: FAO_X_ (X: 5, 10, 12, and 15% Dietary Fe Bioavailability) [49]. The FAO considers three levels of bioavailability of Zn, according to the phytate (Ins*P*_6_) content in the diet: FAO_high_ (Ins*P*_6_/mineral < 5), FAO_moderate_ (Ins*P*_6_/mineral 5–15), FAO_low_ (Ins*P*_6_/mineral > 15) [49]. The EFSA/PRIs for Zn differentiate for levels of Ins*P*_6_ per day (EFSA_300_, 300; EFSA_600_, 600; EFSA_900_, 900; and EFSA_1200_, 1200 mg/day) [52]. ^3^ Adequate fibre intake contribution—Values refer to dietary fibre daily recommendation for adults, calculated based on an intake of 100 g of fresh pasta. Abbreviations: WF (wheat flour-control); and wheat flour-based pastas added with: WQF (white quinoa flour); RQF (red quinoa flour); BQF (black quinoa flour); WQFi (white quinoa fibre); RQFi (red quinoa fibre); BQFi (black quinoa fibre).

## Data Availability

The data presented in this study are available on request from the corresponding author due to ongoing publication and thesis-related work.

## References

[1] Borczak B., Marqués J.M.C., Paredes-López O., Haros C.M. (2023). Structure and Composition of Latin-American Crops. Latin-American Seeds.

[2] Haros C.M., Sanz-Penella J.M. (2017). Food uses of whole pseudocereals. Pseudocereals: Chemistry and Technology.

[3] Mu H., Xue S., Sun Q., Shi J., Zhang D., Wang D., Wei J. (2023). Research progress of quinoa seeds (*Chenopodium quinoa* Wild.): Nutritional components, technological treatment, and application. Foods.

[4] Reguera M., Haros C.M. (2017). Structure and composition of kernels. Pseudocereals: Chemistry and Technology.

[5] Ballester-Sánchez J., Fernández-Espinar M.T., Haros C.M. (2020). Isolation of red quinoa fibre by wet and dry milling and application as a potential functional bakery ingredient. Food Hydrocoll..

[6] Escribano J., Cabanes J., Jiménez-Atiénzar M., Ibañez-Tremolada M., Gómez-Pando L.R., García-Carmona F., Gandía-Herrero F. (2017). Characterization of betalains, saponins and antioxidant power in differently colored quinoa (*Chenopodium quinoa*) varieties. Food Chem..

[7] Tang Y., Li X., Zhang B., Chen P.X., Liu R., Tsao R. (2015). Characterisation of phenolics, betanins and antioxidant activities in seeds of three *Chenopodium quinoa* Willd. genotypes. Food Chem..

[8] Xi X., Fan G., Xue H., Peng S., Huang W., Zhan J. (2024). Harnessing the potential of quinoa: Nutritional profiling, bioactive components, and implications for health promotion. Antioxidants.

[9] Ballester-Sánchez J., Gil J.V., Haros C.M., Fernández-Espinar M.T. (2019). Effect of incorporating white, red or black quinoa flours on free and bound polyphenol content, antioxidant activity and colour of bread. Plant Foods Hum. Nutr..

[10] Alonso-Álvarez A., Haros C.M. (2025). Quinoa fibre isolated by wet milling as a new ingredient for food enrichment: Nutritional value and technological properties. Plant Foods Hum. Nutr..

[11] Haros C.M., Wronkowska M. (2017). Pseudocereal dry and wet milling: Processes, products and applications. Pseudocereals: Chemistry and Technology.

[12] Reynolds A.N., Cummings J., Tannock G., Mann J. (2026). Dietary fibre as an essential nutrient. Nat. Food.

[13] World Health Organization (2023). Carbohydrate Intake for Adults and Children: WHO Guideline.

[14] Anandakumar A., Bhandari M., Singh J. (2025). Micronutrient enriched pasta. Advances in Pasta Technology.

[15] Dziki D. (2021). Current trends in enrichment of wheat pasta: Quality, nutritional value and antioxidant properties. Processes.

[16] Gaikwad P.S., Oswal M., Malini S.H., Supreetha S. (2025). Fiber-enriched pasta. Advances in Pasta Technology.

[17] Li M., Zhu K., Guo X., Brijs K., Zhou H. (2014). Natural additives in wheat-based pasta and noodle products: Opportunities for enhanced nutritional and functional properties. Comp. Rev. Food Sci. Food Saf..

[18] Ballester-Sánchez J., Gil J.V., Fernández-Espinar M.T., Haros C.M. (2019). Quinoa wet-milling: Effect of steeping conditions on starch recovery and quality. Food Hydrocoll..

[19] Association of Official Analytical Chemists (AOAC) (1996). Official Methods of Analysis.

[20] (2016). Food Products—Determination of the Total Nitrogen Content by Combustion According to the Dumas Principle and Calculation of the Crude Protein Content—Part 1 and 2: Cereals, Pulses and Milled Cereal Products.

[21] Association of Official Analytical Chemists (AOAC) (2005). Official Methods of Analysis.

[22] American Association of Cereal Chemist (AACC) (1995). AACC Approved Methods of Analysis.

[23] American Association of Cereal Chemist (AACC) (2000). Approved Methods of Analysis.

[24] Alonso-Álvarez A., Haros C.M. (2025). Revalorization of protein-rich or fibre-rich chia by-products from defatted flours, as nutritional ingredients in the production of fresh pasta. Eur. Food Res. Technol..

[25] Konica Minolta (2002). Chroma Meter CR-400/410 Instruction Manual.

[26] Pathare P.B., Opara U.L., Al-Said F.A.-J. (2013). Colour measurement and analysis in fresh and processed foods: A review. Food Bioprocess Technol..

[27] Zhu K., Kanu P.J., Claver I.P., Zhu K., Qian H., Zhou H. (2009). A method for evaluating Hunter whiteness of mixed powders. Adv. Powder Technol..

[28] Shevell S.K. (2003). Color Appearance. The Science of Color.

[29] Association of Official Analytical Chemists (AOAC) (1990). Official Methods of Analysis.

[30] Sánchez A., Vélez D., Devesa V. (2024). Processes influencing the toxicity of microplastics ingested through the diet. Food Chem..

[31] European Parliament and Council of the European Union (2006). Regulation (EC) No 1924/2006 of the European Parliament and of the Council of 20 December 2006 on nutrition and health claims made on foods. Off. J. Eur. Union.

[32] Espinosa-Solís V., Zamudio-Flores P.B., Tirado-Gallegos J.M., Ramírez-Mancinas S., Olivas-Orozco G.I., Espino-Díaz M., Hernández-González M., García-Cano V.G., Sánchez-Ortíz O., Buenrostro-Figueroa J.J. (2019). Evaluation of cooking quality, nutritional and texture characteristics of pasta added with oat bran and apple flour. Foods.

[33] Petitot M., Boyer L., Minier C., Micard V. (2010). Fortification of pasta with split pea and faba bean flours: Pasta processing and quality evaluation. Food Res. Int..

[34] Jongrattanavit K., Pinkaew P. (2025). Development of nutrient-enriched gluten-free pasta using brown rice flour and green leafy vegetables as natural sources of calcium and dietary fiber. Discov. Food.

[35] Ronda F., Rivero P., Caballero P.A., Quilez J. (2012). High insoluble fibre content increases in vitro starch digestibility in partially baked breads. Int. J. Food Sci. Nutr..

[36] Sudha M.L., Baskaran V., Leelavathi K. (2007). Apple pomace as a source of dietary fiber and polyphenols and its effect on the rheological characteristics and cake making. Food Chem..

[37] Bianchi F., Tolve R., Rainero G., Bordiga M., Brennan C.S., Simonato B. (2021). Technological, nutritional and sensory properties of pasta fortified with agro-industrial by-products: A review. Int. J. Food Sci. Technol..

[38] Atzler J.J., Crofton E.C., Sahin A.W., Ispiryan L., Gallagher E., Zannini E., Arendt E.K. (2024). Effect of fibre fortification of low FODMAP pasta. Int. J. Food Sci. Nutr..

[39] Namir M., Iskander A., Alyamani A., Sayed-Ahmed E.T.A., Saad A.M., Elsahy K., El-Tarabily K.A., Conte-Junior C.A. (2022). Upgrading common wheat pasta by fiber-rich fraction of potato peel byproduct at different particle sizes: Effects on physico-chemical, thermal, and sensory properties. Molecules.

[40] Dey D., Richter J.K., Ek P., Gu B.-J., Ganjyal G.M. (2021). Utilization of food processing by-products in extrusion processing: A review. Front. Sustain. Food Syst..

[41] Itusaca-Maldonado Y.M., Apaza-Humerez C.R., Pumacahua-Ramos A., Mayta Pinto E. (2024). Technological and textural properties of gluten-free quinoa-based pasta (*Chenopodium quinoa* Wild). Heliyon.

[42] Repo-Carrasco-Valencia R., Hellström J.K., Pihlava J.-M., Mattila P.H. (2010). Flavonoids and other phenolic compounds in Andean indigenous grains: Quinoa (*Chenopodium quinoa*), kañiwa (*Chenopodium pallidicaule*) and kiwicha (*Amaranthus caudatus*). Food Chem..

[43] Torres O.L., Lema M., Galeano Y.V. (2021). Effect of using quinoa flour (*Chenopodium quinoa* Willd.) on the physicochemical characteristics of an extruded pasta. Int. J. Food Sci..

[44] Carcea M., Narducci V., Turfani V., Giannini V. (2017). Polyphenols in raw and cooked cereals/pseudocereals/legume pasta and couscous. Foods.

[45] Patras A., Brunton N.P., O’Donnell C., Tiwari B.K. (2010). Effect of thermal processing on anthocyanin stability in foods; mechanisms and kinetics of degradation. Trends Food Sci. Technol..

[46] Vega-Gálvez A., Miranda M., Vergara J., Uribe E., Puente L., Martínez E.A. (2010). Nutrition facts and functional potential of quinoa (*Chenopodium quinoa* willd.), an ancient Andean grain: A review. J. Sci. Food Agric..

[47] Ozturk L., Yazici M.A., Yucel C., Torun A., Cekic C., Bagci A., Ozkan H., Braun H.J., Sayers Z., Cakmak I. (2006). Concentration and localization of zinc during seed development and germination in wheat. Physiol. Plant.

[48] Steadman K.J., Burgoon M.S., Lewis B.A., Edwardson S.E., Obendorf R.L. (2001). Buckwheat seed milling fractions: Description, macronutrient composition and dietary fibre. J. Cereal Sci..

[49] FAO, WHO (2001). Human Vitamin and Mineral Requirements. Report of a Joint FAO/WHO Expert Consultation.

[50] (2015). EFSA Panel on Dietetic Products, Nutrition and Allergies (NDA). Scientific Opinion on Dietary Reference Values for calcium. EFSA J..

[51] (2015). EFSA Panel on Dietetic Products, Nutrition and Allergies (NDA). Scientific Opinion on Dietary Reference Values for iron. EFSA J..

[52] (2014). EFSA Panel on Dietetic Products, Nutrition and Allergies (NDA). Scientific Opinion on Dietary Reference Values for zinc. EFSA J..

[53] Hallberg L., Brune M., Rossander L. (1989). Iron absorption in man: Ascorbic acid and dose-dependent inhibition by phytate. Am. J. Clin. Nutr..

[54] Barbro N., Brittmarie S., Åke C. (1985). Reduction of the phytate content of bran by leavening in bread and its effect on zinc absorption in man. Br. J. Nutr..

[55] Turnlund J.R., King J.C., Keyes W.R., Gong B., Michel M.C. (1984). A stable isotope study of zinc absorption in young men: Effects of phytate and a-cellulose. Am. J. Clin. Nutr..

[56] Gibson R.S. (2006). Zinc: The missing link in combating micronutrient malnutrition in developing countries. Proc. Nutr. Soc..

[57] Ma G., Jin Y., Piao J., Kok F., Guusje B., Jacobsen E. (2005). Phytate, calcium, iron, and zinc contents and their molar ratios in foods commonly consumed in China. J. Agric. Food Chem..

[58] Morris E.R., Ellis R. (1985). Bioavailability of Dietary Calcium. Nutritional Bioavailability of Calcium.

[59] Hurrell R.F. (2004). Phytic acid degradation as a means of improving iron absorption. Int. J. Vitam. Nutr. Res..

